# Quality of life in patients with juvenile arthritis: adalimumab might make a difference

**DOI:** 10.1186/1546-0096-9-S1-P40

**Published:** 2011-09-14

**Authors:** Rina Denisova, Ekaterina Alexeeva, Saniya Valieva, Tatyana Bzarova, Elena Mitenko, Tatyana Sleptsova, Kseniya Isayeva

**Affiliations:** 1 Scientific Centre of Children’s Health of RAMS, Moscow, Russian Federation

## Introduction

Juvenile arthritis (JA) leads to premature impairment of pediatric physical health, psychological and social limitations, that considerably worsen health related quality-of-life of patients. That is why improvement of health related quality-of-life is one of the main aims of treatment of patients with juvenile arthritis.

## Purpose

Evaluate influence of adalimumab therapy on HRQOL of children with different forms of juvenile arthritis.

## Material and methods

There were examined 66 children (median age 10 (3; 16) years). From them there were 44 girls, 23 boys, 23 persons with oligoarticular juvenile idiopathic arthritis (JIA), 30-with polyarticular JIA, 3-with systemic JIA and 10 – with Juvenile Enthesitis related arthritis. 58 (88%) children were treated with adalimumab for more than a year in the dose of 40 mg every 2 weeks Evaluation of HRQOL was performed by the help of questionnaires PedsQL Generic Core Scale (PedsQL GCS), PedsQL Rheumatology Module (PedsQL RM).

## Results

HRQOL of children with JA before the conducted therapy in all scales of questionnaire PedsQL GCS was considerably lower in comparison with population norm (p<0,001). To week 54 of infliximab therapy there was detected statistically reliable increase of HRQOL values in all scales of the questionnaires (p<0,001). By self report of the questionnaires PedsQL RM by the scale “pain and hurt” HRQOL Me increased from 37 to 100, by the scale “daily activities” – from 75 to 100, by the scale “treatment” – from 45 to 74, by the scale “worry” from 45 to 65, by the scale “communication” from 55 to 75. By proxy report from 25 to 100, from 55 to 100, from 30 to 60, from 35 to 62, from 50 to 70, respectively. By self report of the questionnaire PedsQL GCS total score of HRQOL increased from 47 to 79,23, by the scale of physical health – from 43 to 86,15, by the scale of emotional functioning – from 45 to 72,16 and by the scale of social functioning – from 50 to 79,38. By proxy report from 38 to 75,14, from 37 to 81, from 35 to 68,28, from 40 to 72,69, respectively. To the year of receiving the therapy by self report health related quality-of-life differed from population norm only by the scale of social functioning (p<0,01), and by proxy report HRQOL was lower only by the scales of emotional and social functioning (p<0,001).

## Conclusion

Adalimumab administration in pediatric rheumatologic practice increases physical, psychological, social adaptation of patients, allows changing diagnosis of such a severe disabling disease, as JA, stopping steadily progressing disease course and preventing severe disability in such tender age.

